# Free-standing supercapacitors from Kraft lignin nanofibers with remarkable volumetric energy density†
†Electronic supplementary information (ESI) available. See DOI: 10.1039/c8sc04936j


**DOI:** 10.1039/c8sc04936j

**Published:** 2019-01-14

**Authors:** Philipp Schlee, Servann Herou, Rhodri Jervis, Paul R. Shearing, Dan J. L. Brett, Darren Baker, Omid Hosseinaei, Per Tomani, M. Mangir Murshed, Yaomin Li, María José Mostazo-López, Diego Cazorla-Amorós, Ana Belen Jorge Sobrido, Maria-Magdalena Titirici

**Affiliations:** a Queen Mary University of London , School of Engineering and Materials Science , Mile End Road , E1 4NS , London , UK . Email: m.titirici@imperial.ac.uk; b Materials Research Institute , Queen Mary University of London , Mile End Road , E1 4NS , London , UK; c Electrochemical Innovation Lab , Dept. of Chemical Engineering , University College London , Torrington Place , WC1E 7JE , London , UK; d RISE Bioeconomy , Drottning Kristinas väg 61 , Stockholm , Sweden; e University of Bremen , Institute of Inorganic Chemistry and Crystallography , Leobener Straβe 7 , D-28359 Bremen , Germany; f Department of Chemistry , University College London , London WC1H 0AJ , UK; g Departamento de Química Inorgánica e Instituto Universitario de Materiales , Universidad de Alicante , Apartado 99 , 03080 Alicante , Spain; h Imperial College London , Department of Chemical Engineering , South Kensington Campus , SW7 2AZ , UK; i University of Bremen , MAPEX Center for Materials and Processes , Bibliothekstraβe 1 , D-28359 Bremen , Germany

## Abstract

We have discovered a very simple method to address the challenge associated with the low volumetric energy density of free-standing carbon nanofiber electrodes for supercapacitors by electrospinning Kraft lignin in the presence of an oxidizing salt (NaNO_3_) and subsequent carbonization in a reducing atmosphere. The presence of the oxidative salt decreases the diameter of the resulting carbon nanofibers doubling their packing density from 0.51 to 1.03 mg cm^–2^ and hence doubling the volumetric energy density. At the same time, the oxidative NaNO_3_ salt eletrospun and carbonized together with lignin dissolved in NaOH acts as a template to increase the microporosity, thus contributing to a good gravimetric energy density. By simply adjusting the process parameters (amount of oxidizing/reducing agent), the gravimetric and volumetric energy density of the resulting lignin free-standing carbon nanofiber electrodes can be carefully tailored to fit specific power to energy demands. The areal capacitance increased from 147 mF cm^–2^ in the absence of NaNO_3_ to 350 mF cm^–2^ with NaNO_3_ translating into a volumetric energy density increase from 949 μW h cm^–3^ without NaNO_3_ to 2245 μW h cm^–3^ with NaNO_3_. Meanwhile, the gravimetric capacitance also increased from 151 F g^–1^ without to 192 F g^–1^ with NaNO_3_.

## Abstract 2

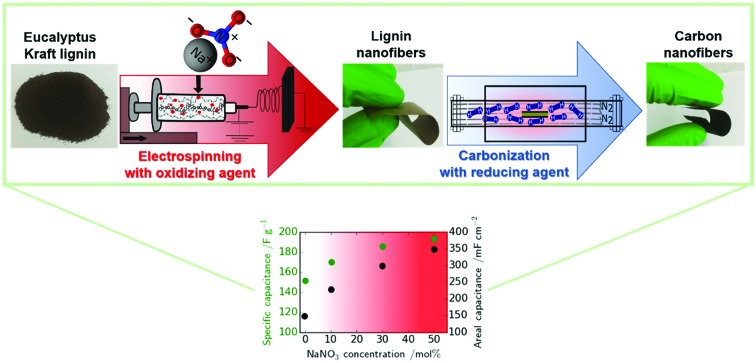
A very simple method to enhance the low volumetric energy density of free-standing carbon nanofiber electrodes.

## Introduction

Carbon fibers are today's materials of choice for hard cases in construction, sporting goods, and electronics as well as for the exterior design in the automotive and aerospace industry. They are mechanically strong (tensile strengths of 3 to 6 GPa, moduli of 200 to 700 GPa) and lightweight, but commonly produced from polyacrylonitrile (PAN) which is an unsustainable precursor made from fossil fuel based acrylonitrile which is polymerized in the presence of toxic solvents and scarce metal catalysts.^1^–^3^ Lignin-based carbon fibers produced *via* melt spinning, electrospinning or centrifugal spinning have recently emerged as sustainable alternatives.^4^–^6^ However, despite many research efforts, lignin-derived carbon fibers cannot compete with the PAN-derived carbon fibers in terms of mechanical properties because lignin does not yield graphitic fibers upon thermal treatment due to its randomly distributed aromatic units which are separated by aliphatic chains.^1^,^7^,^8^ Hence, the tensile strength and moduli for meltspun lignin-derived carbon fibers are significantly lower than those for PAN-derived carbon fibers.^8^,^9^

Another big challenge in lignin-derived carbon fibers is batch to batch reproducibility as lignin is a class of diverse biopolymers, where each lignin varies depending on the parent biomass source, its extraction process and refinery (*e.g.* fractionation).

Here we expand the use of lignin and lignin-derived carbon fibers by preparing porous lignin-based carbon nanofibers (CNFs) by electrospinning which can be used as free-standing and flexible electrodes in supercapacitors (SCs). Our approach is to use eucalyptus Kraft lignin fractionated by sequential solvent extraction with well-defined properties (molecular weight/polydispersity, ash, carbohydrate and inorganic element content) as presented in Tables S1 and S2 in the ESI.† We would like to stress here that in lignin utilisation the reproducibility of the precursor is key in achieving reproducible materials and properties. We developed a new process to produce CNFs by electrospinning of eucalyptus Kraft lignin in a NaOH/NaNO_3_ solution. NaOH dissolves the Kraft lignin and creates micropores during subsequent carbonization. The oxidative salt, NaNO_3_, controls the lignin fiber diameter, and creates additional microporosity and oxygenated groups with pseudocapacitive and wetting effects.^10^ Furthermore, the charge density in the electrospinning solution is increased by NaNO_3_, hence the packing density can be tailored.

Thus, by simply adjusting the amount of NaNO_3_ oxidant in the electrospinning solution both the gravimetric and volumetric energy density of the resulting SCs could be tuned. This represents an elegant and simple approach to produce free-standing and flexible lignin-based CNFs exhibiting tuneable gravimetric and areal capacitance. This can be of great interest for various applications ranging from portable energy devices to automotive industry which require high power and high energy density while building in device flexibility. At the same time, the supercapacitor device will be of low cost since eucalyptus Kraft lignin is a highly underused by-product of the pulp and paper industry.^11^–^13^ In addition, electrospinning is a cost-effective and scalable process to manufacture free-standing and flexible carbon nanofiber mats.^14^–^17^

The literature contains reports on electrospun lignin based carbon fibers for supercapacitors.^18^ Lai *et al.* produced free-standing and mechanically flexible mats from alkali lignin blended with PVA achieving a gravimetric specific capacitance of 64 F g^–1^.^15^ Lignin-derived electrospun carbon fibers were also used as flexible and conductive substrates for MnO_2_ nanowhisker deposition. These composites achieved a specific capacitance of 83.3 F g^–1^ in an organic electrolyte.^14^

Our approach represents a new concept due to its simplicity and tunability. We can tune the fiber porosity and density of the CNF mats by electrospinning in the presence of an oxidizing salt and subsequent carbonization in a reducing atmosphere which creates both micropores (double layer storage) and controlled oxygen surface functional groups (pseudocapacitance/wetting). This results in free-standing CNF mats which are suitable electrodes for the next generation energy storage devices which will be highly flexible and even wearable.^1^,^19^

## Results and discussion

**Fig. 1:**
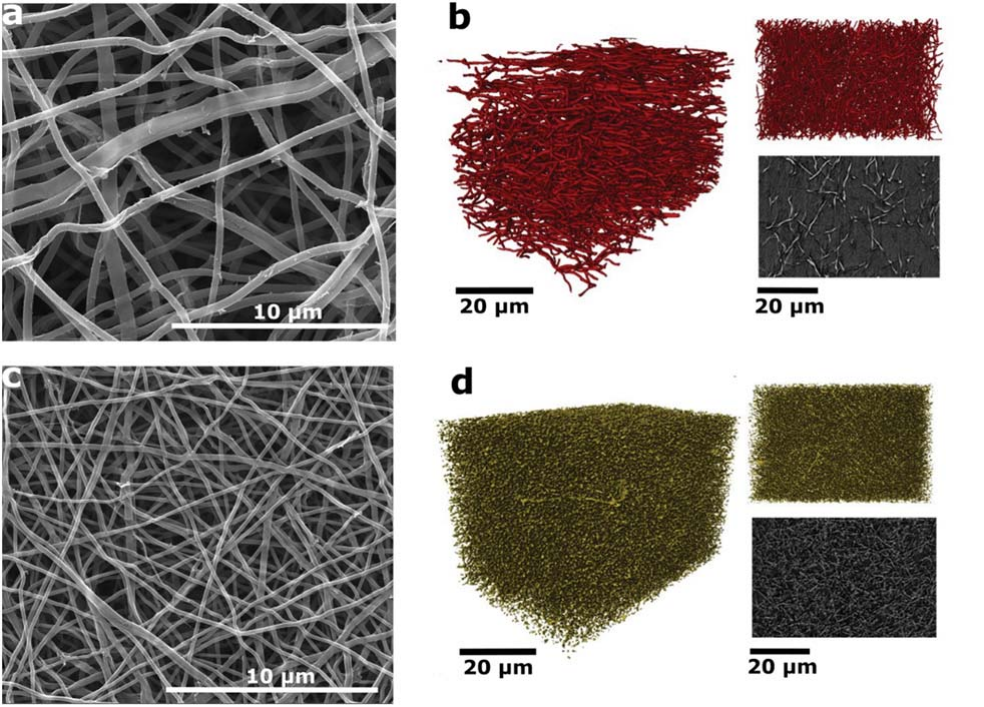
(a) SEM micrograph of the CNF mats with 0 mol% of NaNO_3_; (b) 3D renderings of the reconstructed volume from CT of N0, (upper right) ‘*xy*’ face, the plane of deposition of fibers and (lower right) orthoslice of the greyscale data from the centre of the volume; (c) SEM micrograph of the CNF mats with 50 mol% of NaNO_3_; (d) 3D renderings of the reconstructed volume from CT of N50, (upper right) ‘*xy*’ face, the plane of deposition of fibers and (lower right) orthoslice of the greyscale data from the centre of the volume.

Eucalyptus Kraft lignin (characterization shown in Tables S1 and S2†) solutions (1 M NaOH) with various amounts of NaNO_3_ were electrospun and subsequently carbonized at 800 °C in a reducing atmosphere (5% H_2_ in N_2_) to produce the carbon nanofiber (CNF) mats. These were then used without any binder, conductive additive or additional current collector as free-standing electrodes in symmetric SCs using Swagelok cells. The abbreviations for the samples in the following are based on the amount of NaNO_3_ in the electrospinning solution: N0 (0 mol% of NaNO_3_), N10 (10 mol% of NaNO_3_), N30 (30 mol%) and N50 (50 mol%). The concentration is given with regard to the solvent (NaOH_(aq)_). The conversion to ratios of the weight of polymer to the amount of salt is provided in Table S3.† Scanning electron microscopy (SEM) micrographs of the carbon nanofibers (CNFs) derived from Kraft lignin with various concentrations of NaNO_3_ in the electrospinning solution are shown in Fig. 1 and S2.† It can be observed that the packing density of fibers within the mats rises with increasing NaNO_3_ concentration in the electrospinning solution. This is also confirmed by the average area density of the CNF mats which was determined by weighing electrodes of N0, N10, N30 and N50 with the same dimensions reported in Table 1, the void space between the fibers apparent in sample N0 (Fig. 1a) decreases in N10 (Fig. S2b†) and N30 (Fig. S2c†) and is smallest for N50 (Fig. 1c). The decrease in void space in between the fibers and the resulting increase in packing density is also confirmed by X-ray computer tomography (X-ray CT) carried out for samples N0 and N50 (Fig. 1b and d).

**Table 1:** Average diameters (nm) of the as-spun and carbonized fibers. Additionally, the average area density (mg cm^–2^) of the carbonized samples is given as a measure of the increase in packing density

Sample	Average fiber diameter (as-spun)	Average fiber diameter (carbonized)	Average area density^*a*^ (carbonized)
N0	478 ± 89	331 ± 42	0.51
N10	182 ± 26	163 ± 42	0.69
N30	241 ± 42	201 ± 38	0.77
N50	255 ± 60	188 ± 38	1.03

^*a*^Average value of 10 samples (weight of electrodes with circular shape with *d* = 1.0 cm).

2D imaging *via* SEM/TEM often masks the true three-dimensional properties of porous media. Hence, nano X-ray computed tomography (CT) was used to visualize the carbonized electrospun mats in 3D. This technique has been recently used to image electrospun fibers, highlighting the need for 3D imaging and nano-scale resolution for smaller fibers.^20^–^22^ In Fig. 1b, the 3D rendering and a greyscale *xy* orthoslice of the reconstructed X-ray CT volume of the untreated N0 sample are shown. The *xy* plane shown in the upper right in Fig. 1b is the plane in which the fibers are deposited, with the thickness of the mat growing with time in the *z*-direction. The lab CT system used was capable of producing a pixel resolution of 63.15 nm, providing clear images and good contrast for the N0 fibers. However, upon treatment with NaNO_3,_ the reduction in fiber diameter is large enough (Table 1) that the resolution limits of the CT instrument were reached. Hence, only larger fibers or nodes from the junctions of multiple fibers are visible at this resolution (Fig. 1d). Nevertheless, it is clear from the images of N50 that there is a densification of the mat and a decrease in void space on NaNO_3_ treatment, from 96% in the case of N0 to 76% for N50.

The fiber thinning upon the addition of NaNO_3_ salt in electrospinning solution is attributed to the increase of charge density and thus enhanced elongation of the electrospinning jets.^23^,^24^ The non-linear decrease in average fiber diameter at high salt concentrations was attributed to an excess of charge density on the collector and hence incomplete splitting of the electrospinning jet.^25^,^26^ The effect of various amounts of NaNO_3_ on the as-spun lignin fibers can be seen in Fig. S1.†

**Fig. 2:**
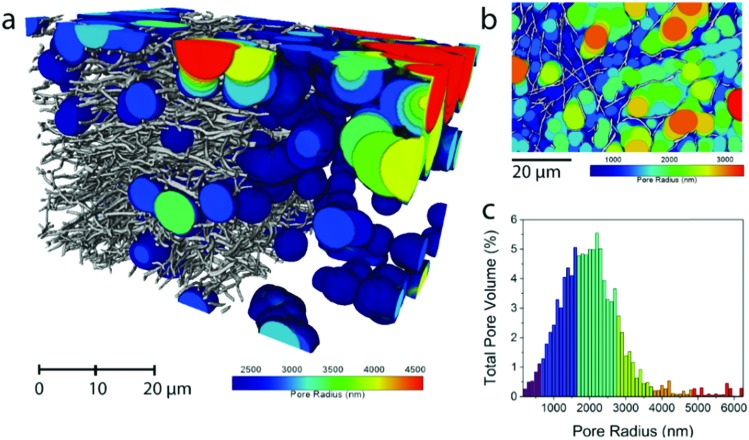
Pore size distribution in the pore phase of the carbonized N0 sample. For clarity, the smaller pores have been removed in (a) and a 2D representation of all the calculated pore radii is shown in (b). The sphere radius was dilated by 100 nm at a time and the histogram of total pore volumes of each sphere radius is shown in (c).

The average fiber diameter for sample N0 was sufficient to perform a continuous pore size distribution in three dimensions on the N0 volume, using a local thickness method calculating the total pore volume that can be filled with spheres of increasing radius (Fig. 2). This yields the 3D porosity of the CNF network. The pore size distribution (Fig. 2c) shows that the voids between the fibers are extremely big (1000–3000 nm) compared to the fiber diameter (331 nm). This reveals the need for densification of electrospun nanofiber mats to make them (space-)efficient electrodes in future devices.

**Fig. 3:**
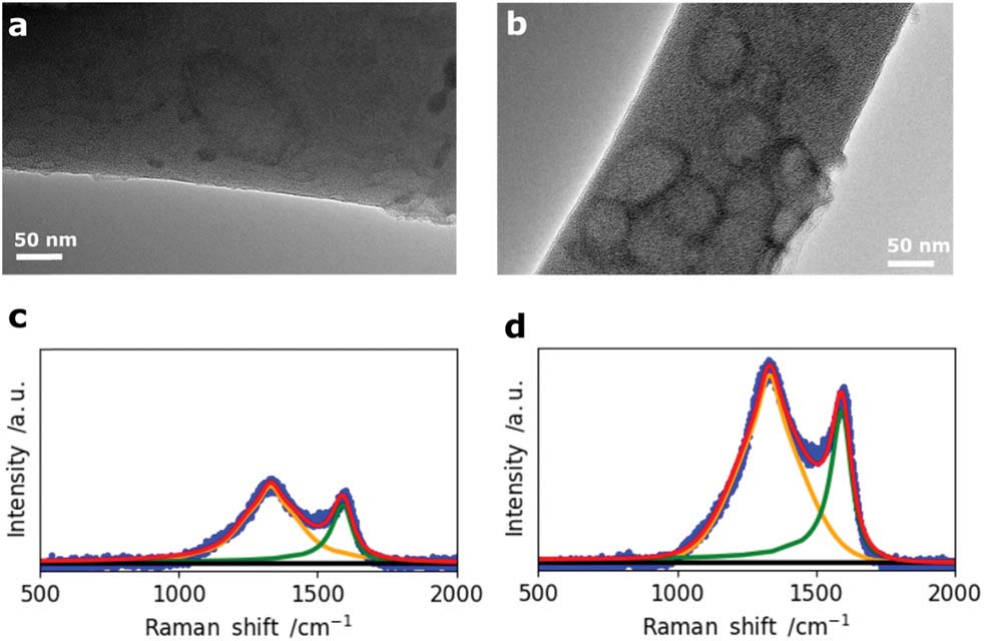
TEM micrographs of (a) N0 and (b) N50 and corresponding Raman spectra of (c) N0 and (d) N50.

In addition to the morphological 2D and 3D analysis of the entire nanofiber network, the change in single fiber morphology was investigated by Transmission Electron Microscopy (TEM). The TEM micrographs of N0 and N50 in Fig. 3a and b show that the fibers are mainly composed of disordered carbon which is also confirmed by the Raman spectra (Fig. 3c and d). However, N50 exhibits ring-like domains on the fiber surface. The X-ray photoelectron spectroscopy (XPS) data given in Table 2 clearly indicate negligible amounts of Na and N in all samples which means the features seen on the fibers are not salt impurities (*e.g.* remaining NaNO_3_). Instead, these ring-like domains might indicate where the activating salt was located before decomposition which led to an enhanced ablation and rearrangement of the carbon matrix in this region. The Raman spectra of N0 and N50 show both D- and G- bands, indicating disordered carbon structures.^27^–^29^

**Table 2:** Overview of the surface chemistry. C, O, N, S and Na content (wt%) of the CNFs based on XPS and O content (wt%) from TPD results

Sample	C	O (TPD)	O	N	S	Na
N0	90.1	2.9	8.9	0.3	0.5	0.2
N10	91.5	3.2	7.0	0.8	0.3	0.3
N30	85.7	3.3	13.1	0.6	0.4	0.2
N50	86.0	4.5	12.8	0.7	0.2	0.2

The crystallite sizes were calculated from the intensity ratios between the D- and G- bands using the method reported by Herdman *et al.*^29^,^30^ The fitting results together with the Raman spectra of all four samples are given in Fig. S5 and Table S4.† There is no significant difference in the crystallite sizes (*L*_a_) among the four CNF samples with *L*_a_ values between 6.3 and 7.0 nm indicating that it is not affected by changes in porosity, morphology and the chemical composition at the same carbonization temperature of 800 °C. The surface chemical composition of the fibers was investigated by temperature programmed desorption (TPD) and X-ray photoelectron spectroscopy (XPS). Hereby, it is clear that the main type of functional groups are oxygen groups as it was started from an oxygen rich precursor (eucalyptus Kraft lignin, Table S1†) which was further oxidized (NaNO_3_) and subsequently reduced (5% H_2_ in N_2_ at 800 °C).

**Fig. 4:**
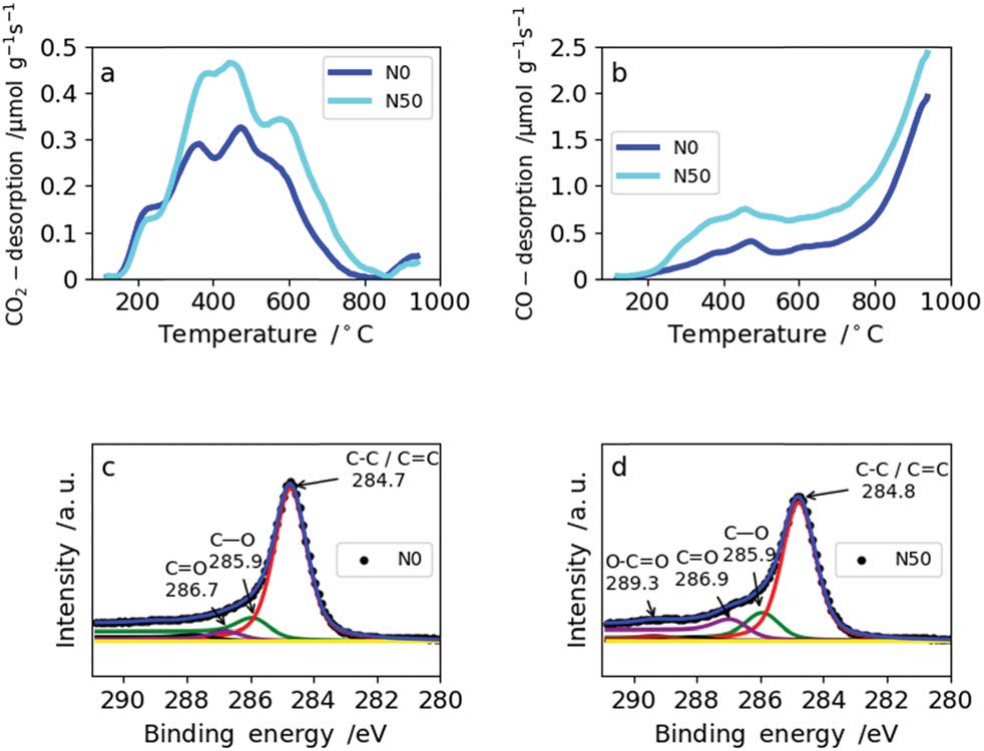
TPD spectra for N0 and N50: (a) CO_2_ desorption rates from TPD, (b) CO desorption from TPD, XPS spectra for N0 and N50: (c) N0 and (d) N50.

The amount and type of oxygen groups are of great importance for the performance of SC electrodes.^31^ It was shown that carbons with basic oxygen functional groups exhibit superior performance to carbons with acidic groups in basic electrolytes (*e.g.* 6 M KOH).^32^ As shown in Table 2, the total oxygen content determined by TPD increases only slightly with increasing amount of NaNO_3_. This is explained by the reducing atmosphere (5% H_2_ in N_2_) during carbonization which effectively removes the oxygen functionalities arising from NaNO_3_ decomposition. Consequently, the total amount of oxygen increases only from 2.9 wt% in N0 to 4.5 wt% for N50 (Table 2). From TPD it is also possible to derive the type of oxygen groups present on the carbon surface based on the gas desorbed: CO_2_ desorption (Fig. 4a) at low temperatures arises from less favorable acidic groups (*e.g.* carboxylic, lactones and anhydrides), whereas basic functional groups give rise to CO desorption (*e.g.* ethers and hydroxyl groups) (Fig. 4b). Both N0 and N50 show higher CO desorption rates than CO_2_ desorption. In N0, the ratio of CO/CO_2_ is 3.1, whereas in N50 it equals 3.7. Hence, the addition of NaNO_3_ with subsequent reduction increases the amount of more favourable basic oxygen groups in the CNFs.

The amount of oxygen groups determined by XPS is also given in Table 2. It shows a slightly lower oxygen content for N10 (7.0 wt%) compared with N0 (8.9 wt%) which is explained by the fact that with the addition of a small amount of NaNO_3_ the porosity and fiber morphology are modified significantly. These changes lead to shadowing of the surface oxygen in narrow micropores which cannot be probed by XPS due to its low penetration depth.^33^ This explains the differences between XPS and TPD in assessing the total oxygen content. The types of oxygen functionalities from the deconvolution of the C1s XPS profiles of N0 and N50 are given in Fig. 4c and d, respectively. From these, it is clear that C–O and C=O groups are predominant in N0 and N50 and carboxylic groups are only minor. TPD and XPS analysis show that the overall amount of mainly basic oxygen groups is enhanced in N50 compared with N0. Furthermore, the XPS data indicate that the CNF surface is free from any residual salts which can remain from the use of NaOH as the electrospinning medium, NaNO_3_ addition or residual inorganics in Kraft lignin. Additionally, it also indicates a very low sulfur content in the carbon fibers which is a result of extensive bio-refinery of the lignins separated by the Lignoboost process and refined *via* a sequential solvent extraction process.^5^,^34^,^35^ The deconvolutions of the C1s and O1s XPS profiles of all four samples are given in Fig. S6 and S7.† Furthermore, the quantification of CO_2_ and CO desorption from TPD can be found in Table S5.†

**Fig. 5:**
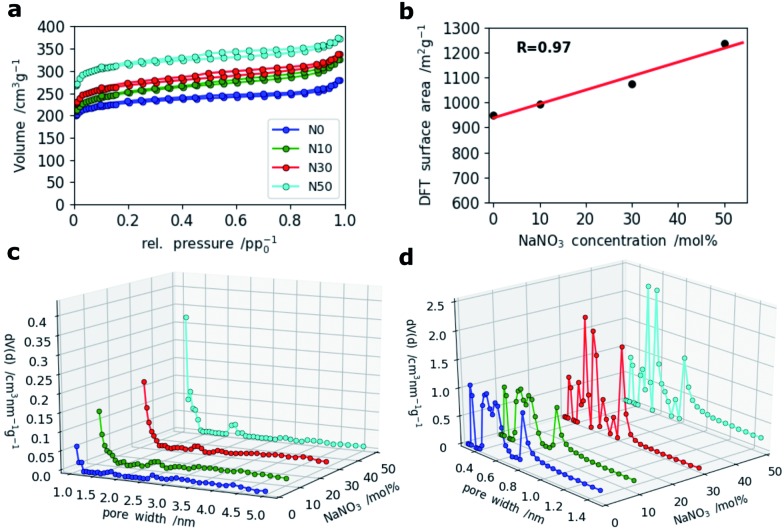
(a) N_2_ sorption isotherms of all four samples, (b) correlation of DFT-derived surface area and NaNO_3_ content, (c) PSDs calculated by DFT (QSDFT (slit pore/cylindrical pore) model) based on the adsorption isotherms of N_2_ and (d) PSDs from CO_2_ adsorption measurements (NLDFT, Non-Localized DFT method).

For SCs, the presence of accessible micropores for reversible ion sorption at the interface between the electrode and electrolyte is crucial. Especially, pores with widths below 1 nm were shown to give a boost in capacitance.^36^ To analyze the porosity of the CNF mats with various amounts of NaNO_3_ in the electrospinning solution, N_2_ and CO_2_ gas sorption measurements were applied. The resulting isotherms of the N_2_-sorption experiments are shown in Fig. 5a. It clearly indicates that microporosity increases with the amount of NaNO_3_ added in the electrospinning solution which is also confirmed by a linear correlation coefficient of 0.97 between the specific surface area and NaNO_3_ content (Fig. 5b). The pore size distributions (PSDs) calculated by Quenched Solid State Density Functional Theory (QSDFT) show that the addition of the oxidizing agent, NaNO_3_, creates slightly bigger pores around 1.0 and 1.4 nm. For the sample N50 even a small hump at around 2.2 nm is observed in the PSD (Fig. 5c).

A closer look at the PSDs within the narrow micropore region using CO_2_ adsorption (Fig. 5d) shows that while N0 and N10 have similar (ultra-)microporous regions (<1 nm), the PSDs for N30 and N50 within this domain are markedly different showing an increase in pore volume between 0.4 and 0.6 nm as indicated by two sharp and distinct peaks at 0.5 and 0.6 nm. This indicates that even at high salt concentration no agglomeration and hence formation of enlarged pores takes place, but rather a fine and homogeneous distribution of activating salt translates into enhancement of microporosity.

**Scheme 1:**
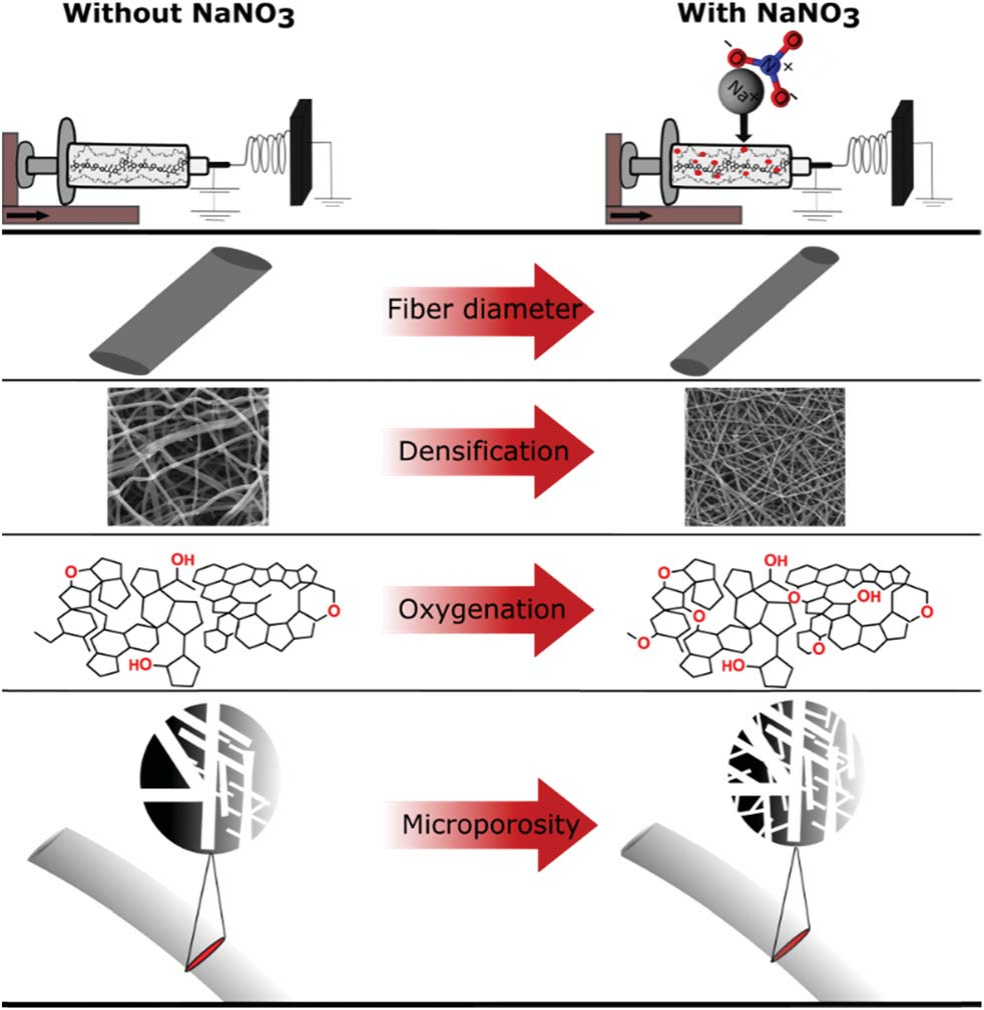
Summary of effects of NaNO_3_ in the electrospinning solution determined by various characterization techniques.

The oxidization by addition of NaNO_3_ and subsequent reduction of the lignin-derived CNFs results in an increase in electrode density as shown by CT and SEM, controlled oxygen functionalization shown by TPD and XPS and an increase in microporosity analysed by gas sorption measurements (Scheme 1). This will have a great impact on the overall performance of the free-standing CNF mats in symmetric supercapacitors. The testing of the CNF mats as free-standing electrodes in symmetric two electrode SCs was carried out in Swagelok cells using a basic aqueous electrolyte.

**Fig. 6:**
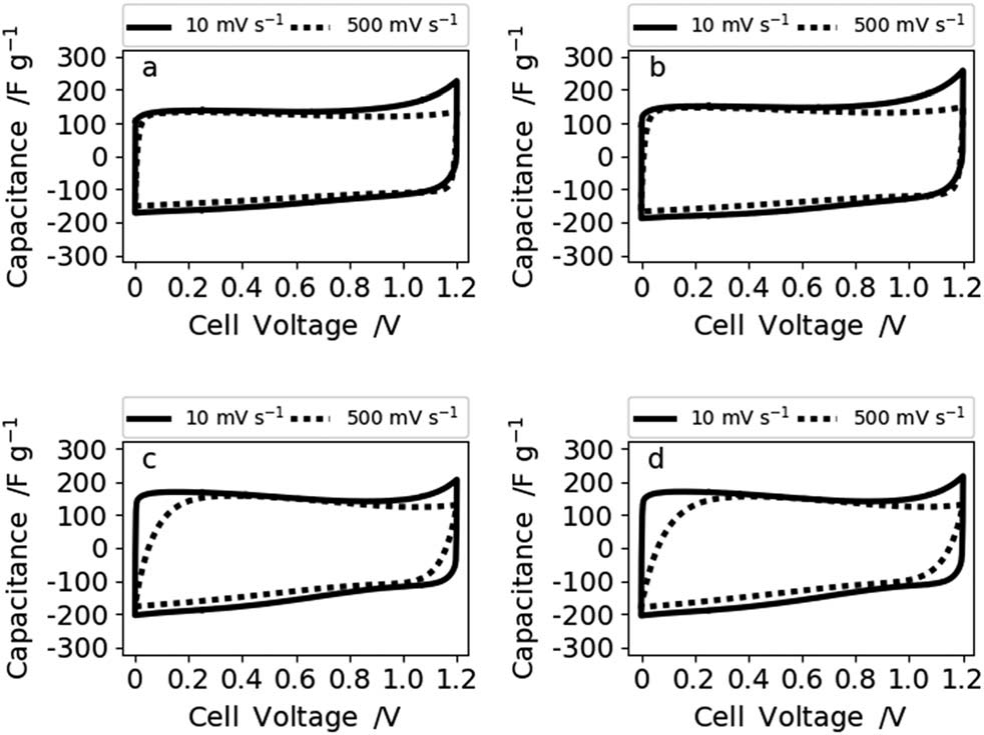
Voltammograms at 10 and 500 mV s^–1^ of (a) N0, (b) N10, (c) N30, and (d) N50 measured in 6 M KOH.

To evaluate the electrochemical performance of our free-standing carbon mats from Kraft lignin in supercapacitors, we have recorded cyclic voltammograms (CVs) in 6 M KOH at various scan rates in two electrode cells and a three-electrode set-up (Fig. S9†). These reveal a similar behavior of all four samples for low (10 mV s^–1^) (Fig. 5) and mid-level (50 mV s^–1^) scan rates (Fig. S8†). However, the CVs of N30 and N50 show a slight curvature and hence higher capacitance values at low scan rate (10 mV s^–1^) which arise from the contribution of the oxygen functional groups.^10^,^37^ A reaction mechanism for the contribution of oxygen groups in alkaline media remains to be elucidated. Increased hydrophilicity and ion affinity are the two contributions most commonly described for oxygen rich carbons tested in alkaline media.^10^,^37^ At high and very high scan rates of 500 mV s^–1^ (Fig. 6) and 1 V s^–1^ (Fig. S8†), N0 and N10 present a rectangular shape of the voltammograms, whereas N30 and N50 exhibit a deformed shape which indicates a lower diffusion rate of ions in the (ultra-)micropores.^38^

**Fig. 7:**
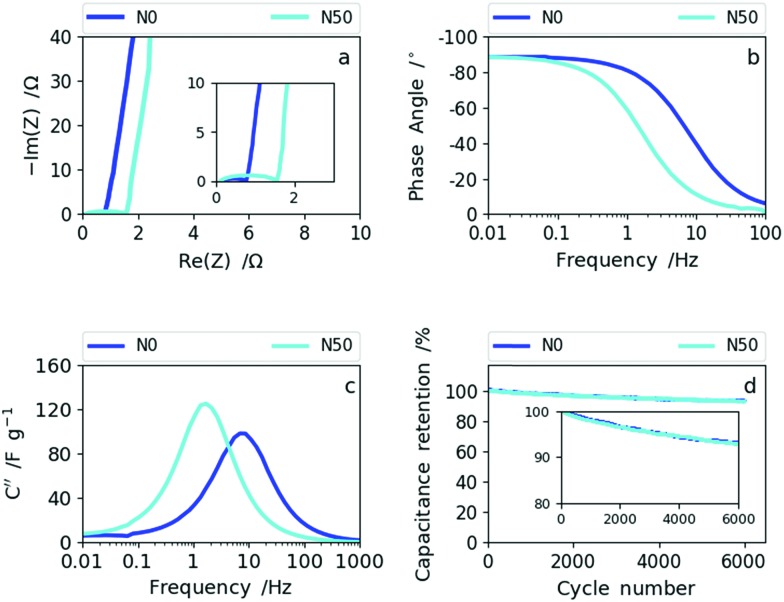
(a) The Nyquist plots of N0 and N50, (b) Bode phase angle plots of N0 and N50, (c) imaginary capacitance *vs.* frequency dependencies of N0 and N50 and (d) cyclability data of N0 and N50.

Therefore, the samples synthesized with no or low NaNO_3_ content (N0 and N10) reveal a better rate capability compared with N30 and N50. This is also supported by the impedance data shown for sample N0 (high rate capability) and N50 (lower rate capability) in Fig. 7.

The Nyquist plot in Fig. 7a shows an enlarged semi-circle for N50 compared to N0 as a result of the increase in oxygen functional groups and the rise in (ultra-)micropores and packing density (decrease in macroporosity).^39^,^40^ In the Bode phase plot (Fig. 7b) the frequency responses of N0 and N50 show that N0 preserves the ideal value of –90° (capacitive) towards higher frequencies indicating higher rate capability than N50. The imaginary capacitance (*C*′′) plotted as a function of frequency confirms this observation (Fig. 7c). The maximum imaginary capacitance is located at the so-called relaxation frequency, *f*_R_, determining the characteristic time constant according to *τ* =(2π*f*_R_)^–1^. In general, the lower the time constant the higher the rate capability.^41^ For N0, *f*_R_= 7.7 Hz and for N50, *f*_R_= 1.7 Hz, hence N0 shows a smaller time constant of 0.02 s than N50 (0.095 s).

The combined CV and impedance analysis reveal clearly that the increase in oxygen groups in N50, the enhancement of (ultra-)microporosity and the densification of the nanofiber network give rise to lower rate capability, but higher capacitance values at low rates. Lower rate capabilities due to oxygen functionalization and due to pores smaller than 1 nm were also reported in previous studies on carbon based supercapacitors.^25^,^42^–^45^ The capacitance retention of 92.5% after 6000 cycles for N50 and N0, which is shown in Fig. 7d (and Fig. S10†), indicates good cyclability supporting the previous observations that basic oxygen functional groups are stable in basic electrolytes.^32^

**Fig. 8:**
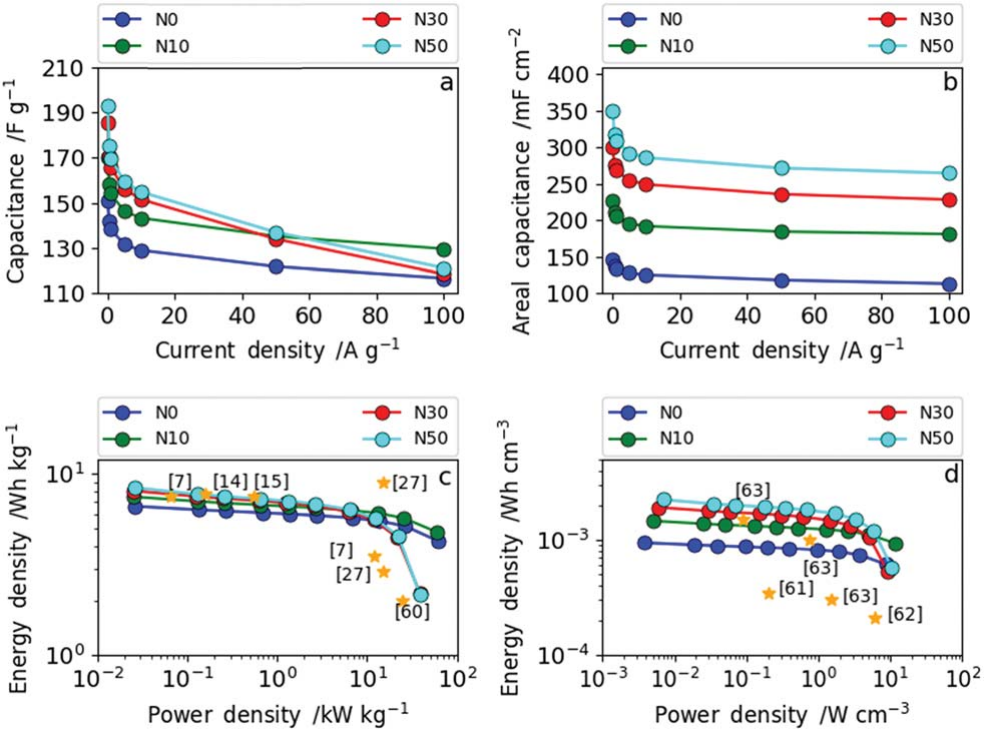
(a) Dependence of specific gravimetric capacitance on current density for all four samples; (b) dependence of specific areal capacitance on current density for all four samples; (c) Ragone plot in gravimetric measures and (d) Ragone plot in volumetric measures.

The rate capability was investigated in more detail by galvanostatic charge–discharge measurements (GCD) at different current densities. In Fig. 8a and b the dependence of (a) specific gravimetric and (b) areal capacitance values on various current densities are presented. The direct comparison of the gravimetric and areal capacitance reveals the role of densification. In Fig. 8a, all results are normalized by the electrode weights (F g^–1^) meaning the densification of the CNF electrode is not taken into account. Hence, the trend already derived from cyclic voltammetry and impedance spectroscopy can also be seen from GCD (Fig. 8a). The samples produced with high amounts of salt (N30 and N50) show lower rate capability than the ones with no or low amounts of NaNO_3_ (N0 and N10). More explicitly, at current densities of up to 50 A g^–1^ (medium rate) the specific gravimetric capacitance increases with increasing amount of NaNO_3_ and a maximum gravimetric capacitance of 192 F g^–1^ at 0.1 A g^–1^ was obtained for N50. At high current densities (charge and discharge rates) exceeding 50 A g^–1^, N10 with a low amount of surface oxygen and moderate increase in (ultra-)microporosity and packing density shows excellent rate capability. Through determination of the areal capacitance the positive effect of fiber network densification can be taken into account. Consequently, the specific areal capacitance values (mF cm^–2^) show even more pronounced differences among the samples (Fig. 8b). At low rates, the areal capacitance is more than doubled from 147 mF cm^–2^ (N0) to 350 mF cm^–2^ (N50). At high rates this leap in areal capacitance is preserved as the increase in packing density, oxygen functionality and (ultra-)microporosity are all factored in. The full cell capacitances (based on the total amount of carbon in the cells) can be derived by dividing the given capacitance values by the constant factor 4.

The Ragone plots in gravimetric and volumetric measures, shown in Fig. 8c and d, reveal the tunability between high power and high energy density based on the NaNO_3_ content in the electrospinning solution. Additionally, references to recent literature reports are given in the same figure to illustrate where we stand in relation with the state of art. N30 and N50 contain increased oxygen functional groups, have (ultra-)microporosity and have a higher fiber packing density operating as high energy density materials (up to 8.4 Wh kg^–1^) at low power (2.6 W kg^–1^). N0 and N10 contain low amounts of surface oxygen and less (ultra-)micropores and have a lower packing density. Consequently, N0 and N10 operate as high power density materials (up to 60 kW kg^–1^).

The same trend as for the gravimetric energy and power densities can be observed in terms of volumetric energy density (Fig. 8d). N10 exhibits a remarkably high power density of 11.6 W cm^–3^ with an energy density of 14.7 × 10^–4^ Wh cm^–3^, whereas N50 delivers the highest energy density with 22.5 × 10^–4^ Wh cm^–3^ and a maximum power density of 10.3 W cm^–3^. As shown in Fig. 8d, the samples N30 and N50 with a high degree of fiber network densification exhibit excellent performance compared to all recent literature reports in terms of volumetric energy and power density. Simply by varying the amount of NaNO_3_ in the electrospinning solution and subsequent reduction (5% H_2_ in N_2_) during carbonization the resulting electrode specifications can be tailored towards high energy (high NaNO_3_ content) or high power (low NaNO_3_ content) applications.

## Experimental

### Preparation of lignin-derived CNF mats

The precursor solutions for electrospinning contained polyethylene oxide (Sigma-Aldrich) with an average molecular weight of 600 000 g mol^–1^, PEO, and eucalyptus Kraft lignin with a molecular weight of 1900 g mol^–1^ and a polydispersity index of 1.5 (Rise AB, Sweden) in a PEO/lignin weight ratio of 0.14 dissolved in 1 M NaOH. More detailed information on the Kraft lignin used is given in the ESI (Tables S1 and S2).† The total amount of polymer in solution was 12 wt%. For oxidation 10, 30 and 50 mol% of NaNO_3_ with respect to the solvent were added to the polymer solution in the form of 0.7 g ml^–1^ NaNO_3(aq)_ solution. The resulting NaNO_3_/lignin ratios are given in the ESI in Table S3.† The samples prepared from the various solutions are abbreviated according to the amount of NaNO_3_ in solution as N0, N10, N30 and N50. These solutions were stirred for 3 hours and then stored in sealed glass vials overnight without stirring before electrospinning. For electrospinning, a plastic syringe (HSW, 10 ml) was loaded with 4 ml of the electrospinning solution. The syringe was discharged at a rate of 1 ml h^–1^ with a syringe pump (Pump 33 DDS Dual Drive System, Harvard Apparatus, USA). Additionally, a high voltage between the syringe needle (gauge = 23) and the collector, which was an aluminum foil (24 × 24 cm), was applied with a transformer (Glassmann High Voltage Inc., USA). In order to control the humidity, electrospinning was carried out in a glovebox (Plas-labs, USA). The parameters adjusted for electrospinning are shown in Table S7.† The parameters for electrospinning were refined so that a stable Taylor cone during the electrospinning process could be established. Additionally, it needs to be mentioned that the electrospinning parameters for all samples are very similar, which means that the comparability among the samples is ensured and hence all differences between the various fiber mats discussed in the following can be attributed mainly to the addition of various amounts of NaNO_3_. After electrospinning, the as-spun lignin fiber mats were converted to carbon nanofiber mats in a one-step thermal treatment by direct carbonization^46^ in forming gas (5% H_2_ in N_2_) without the need for a separate stabilization step due to PEO which hydrogen-bonds with the lignin in solution.^47^ For direct carbonization, the fiber mats were heated to 800 °C for 2 h at a heating rate of 3 °C min^–1^. Finally, the carbonized mats were washed in a water bath at 80 °C for 1 h and subsequently dried overnight at 120 °C to remove salts and residual activation agent.

### Morphological characterization – SEM, X-ray CT and TEM

The morphology of the fibers and nanofiber mats was characterized using a FEI Inspect F scanning electron microscope (SEM) using an accelerating voltage of 20 kV. Fiber diameter distributions were generated from SEM images using the ImageJ software package (U.S. National Institutes of Health).^48^ The diameters are reported as the mean and standard deviation based on measurements of 100 fibers.

X-ray nano-CT was performed on the point of triangular samples of the mats glued to a pin, and by selecting a scanning region that was fully internal, but as close to the point of the triangle as possible. A lab based nano-CT instrument (Zeiss Xradia Ultra 810, Carl Zeiss Inc.) was used in phase contrast mode, containing a rotating Cr anode source set to an accelerating voltage of 35 kV. The beam was quasi-monochromatized at the Cr-Kα emission line of 5.4 keV by a capillary condenser, illuminating the sample with a hollow cone beam focused on the sample, with a Fresnel zone plate as the objective element creating a magnified image on a 1024 × 1024-pixel CCD detector. The insertion of a Au phase ring in the back focal plane of the objective shifts the undiffracted component of the beam, resulting in negative Zernike phase contrast (more details of which can be found in the paper of Tkachuk *et al.*^49^). Large field-of-view mode with a pixel binning of 1 was used, resulting in a pixel size of *ca.* 63 nm and a field-of-view of *ca.* 65 μm. The sample was rotated through 180° with radiographs collected at discrete angular intervals amounting to 1601 projections, each of 40 s exposure. The radiographic projections were then reconstructed with proprietary reconstruction software (XMReconstructor, Carl Zeiss Inc.) using a parallel beam reconstruction algorithm. The grey-scale reconstructed volume was then segmented into a binary image using Avizo Fire software (Thermo Fisher Scientific, Waltham, MA, USA) to designate pixels as either ‘fiber’ or ‘pore’ materials by thresholding based on their greyscale value on a cropped dataset resulting in a volume of 650 × 650 × 1000 pixels, or slightly more than 10 × 10^4^ μm^3^. Avizo Fire was also used for 3D visualization. The ‘Beat’ plug in of ImageJ^50^ was used to calculate the continuous pore size distribution on a binned dataset (325 × 325 × 500 pixels), and also applied to the fiber phase for the original dataset.

TEM images were recorded with a JEOL 2010 operated at 200 kV.

### Spectroscopic characterization – Raman

Raman spectra were recorded on a LabRAM ARAMIS (Horiba Jobin Yvon) Micro-Raman spectrometer equipped with a laser working at 633 nm and less than 20 mW. The use of a 50× objective (Olympus) with a numerical aperture of 0.75 provides a focus spot of about 1 μm diameter when closing the confocal hole to 200 μm. Raman spectra were collected in the range between 500 cm^–1^ and 2500 cm^–1^ with a spectral resolution of approximately 2 cm^–1^ using a grating of 1800 grooves per mm and a thermoelectrically cooled CCD detector (Synapse, 1024 × 256 pixels). The spectral positions were calibrated against the Raman band of Si (520.7 ± 0.4 cm^–1^) before and after the measurements. The linearity of the spectrometer was calibrated against the emission lines of a neon lamp.

### Chemical characterization – XPS and TPD

The surface chemistry of the carbon fibers was analyzed by X-ray photoelectron spectroscopy (XPS) using a Thermo Scientific Kα spectrometer with an Al Kα monochromatic source (1486.6 eV) and a multidetection analyser under a residual pressure of 5 × 10^–8^ mbar. Temperature programmed desorption (TPD) experiments were carried out in a TGA-DSC instrument (TA Instruments, SDT Q600 Simultaneous) coupled to a mass spectrometer (Thermostar, Balzers, GSD 300 T3) by heating the samples (approx. 4 mg) up to 950 °C (heating rate: 20 °C min^–1^) in a helium atmosphere (flow rate: 100 ml min^–1^). The thermobalance was purged for 2 hours in a helium atmosphere prior to the heating of the sample. The calibration of the equipment for H_2_O, CO and CO_2_ evolved gases was carried out using the decomposition of a calcium oxalate (99.999%, Sigma Aldrich) standard.

### Porosity – gas sorption measurements

The specific surface areas and pore size distributions of the carbonized fiber mats were determined by N_2_ and CO_2_ adsorption/desorption measurements at –196 °C and 0 °C, respectively, using an Autosorb iQ-C (Quantachrome Instruments, USA). Samples were degassed at 200 °C for 16 h under vacuum (2.7×10^–3^mbar) before analysis. Pore size distributions (PSDs) were calculated by Quenched Solid State Density Functional Theory (QSDFT), based on the slit pore/cylindrical pore model and Non-Localized Density Functional Theory (NLDFT).

### Electrochemical measurements

The synthesized Kraft lignin-derived CNF mats were tested as electrodes in symmetric supercapacitor cells. Hereby, all measurements were performed in a two-electrode set-up consisting of two CNF electrodes in circular shape with a diameter of 1 cm. The cells were assembled in a Swagelok-type cell and connected to a VSP-potentiostat (Biologic, France). The electrolyte was 6 M KOH and a standard filter paper was used as a separator (Whatman, GE Healthcare Life Sciences, UK, Grade GF/D). Before the actual measurements, the cells were galvanostatically charged and discharged for 500 cycles at a current density of 5 A g^–1^ to ensure sufficient wetting of the electrodes with the electrolyte. Subsequently, cyclic voltammetry was performed at different scan rates and galvanostatic charge–discharge with potential limitation at different current densities was carried out. Impedance spectroscopy was conducted at open circuit potential and with an amplitude of 5 mV in a frequency range of 10 mHz to 500 kHz. Finally, capacitance retention was measured at a scan rate of 5 A g^–1^ for 6000 cycles. Additionally, three-electrode measurements were carried out where a platinum counter electrode, 6 M KOH as the electrolyte and carbon fiber samples as the working electrode were used.

## Conclusions

We have successfully tuned the specific gravimetric capacitance of Kraft lignin-derived CNFs from 151 to 192 F g^–1^ in 6 M KOH at a low current density of 0.1 A g^–1^*via* a two-step procedure of oxidative electrospinning and reducing carbonization. Our method allows doubling of the loading per electrode *via* fiber network densification with the addition of NaNO_3_ which consequently allows the areal capacitance to more than double from 147 mF cm^–2^ in the absence of NaNO_3_ to 350 mF cm^–2^ with NaNO_3_ with good capacitance retention of 92.5% after 6000 cycles. Thus, the low volumetric energy density normally associated with CNF mats could be greatly improved from 949 μW h cm^–3^ to 2245 μW h cm^–3^. To the best of our knowledge, this is among the highest volumetric energy densities obtained for free-standing CNF electrodes. Finally, the two-step procedure of oxidative electrospinning and reducing carbonization introduced here can easily be expanded by varying the type/amount of oxidizing/reducing agent and gives the opportunity to tailor the rate capability of biomass-derived carbons for next generation SCs in order to meet the demands of both high energy and power densities in energy storage in a free-standing electrode design.

## Conflicts of interest

There are no conflicts to declare.

## Supplementary Material

Supplementary information
