# Supplementary material for: Free-standing supercapacitors from Kraft lignin nanofibers with remarkable volumetric energy density
Source: Chem Sci. 2019 Jan 14;10(10):2980–8. doi: 10.1039/c8sc04936j (PMC6427945; doi:10.1039/c8sc04936j)
Supplement: Supplementary file 1 [file SC-010-C8SC04936J-s001.pdf]

Electronic Supplementary Material (ESI) for Chemical Science.
This journal is © The Royal Society of Chemistry 2019

Free-standing Supercapacitors from Kraft Lignin Nanofibers with Remarkable Volumetric Energy Density

Philipp Schlee[ab], Servann Herou[abh], Rhodri Jervis[c], Paul R. Shearing[c], Dan J. L. Brett[c], Darren Baker[d], Omid Hosseinaei[d], Per Tomani[d], M. Mangir Murshed[ei], Yaomin Li[f], Maria José Mostazo-López[g], Diego Cazorla-Amorós[g], Ana Belen Jorge Sobrido[ab], Maria-Magdalena Titirici[†abh].

Supplementary information

Molecular weight determination was performed by size exclusion chromatography (SEC). The SEC system consisted of a pre-column followed by two analytical columns, PSS MCX 10^2 and 10^4 nm, connected in series. The mobile phase was of a pH 12 buffer solution (NaOH and NaH_2PO_4*H_2O). The flow rate was 0.5 ml min^{-1}. The samples were dissolved in NaOH, pH 13.5 and diluted to suitable concentrations with the mobile phase. Prior to injection, the samples were filtered (cellulose acetate syringe filter, 0.20 µm). The injection volume was 100 µl. Detection was performed using a UV detector (Knauer K-2501). The UV absorbance was measured at 280 nm. The SEC system was calibrated using sulfonated polystyrene standards with molecular weights ranging from 891 to 780 000 g mol^{-1}.

Molecular weight of 1900 g mol^{-1} and PDI of 1.5.

Tab. S1 Elemental analysis of hardwood Kraft lignin precursor.

Element	Content /wt%	Standard dev /wt%
C	62.21	0.07
H	5.56	0.01
N	0.17	0.01
S	2.06	0.01
O	30.0	n.a.

The ash content was determined to be 0.3 wt%.

Tab. S2 Results of Inductively Coupled Plasma-Optical Emission Spectroscopy (ICP-OES) of hardwood Kraft lignin precursor.

Element	(mg/kg DS)
Al	23
Ba	1
Ca	69
Cu	5.5
Fe	43
K	114
Mg	22
Mn	2.5
Na	556
P	<1,25
S	23083
Si	8.2
Zn	1.8
Total	23,929
Total-S	846

Information on the $NaNO_3$/polymer weight ratios in the spinning solutions:

Tab. S3 $NaNO_3$/polymer weight ratios in the spinning solutions.

Sample	$NaNO_3$/ mol %	$NaNO_3$/polymer
N0	0	0.000
N10	10	0.059
N30	30	0.179
N50	50	0.299

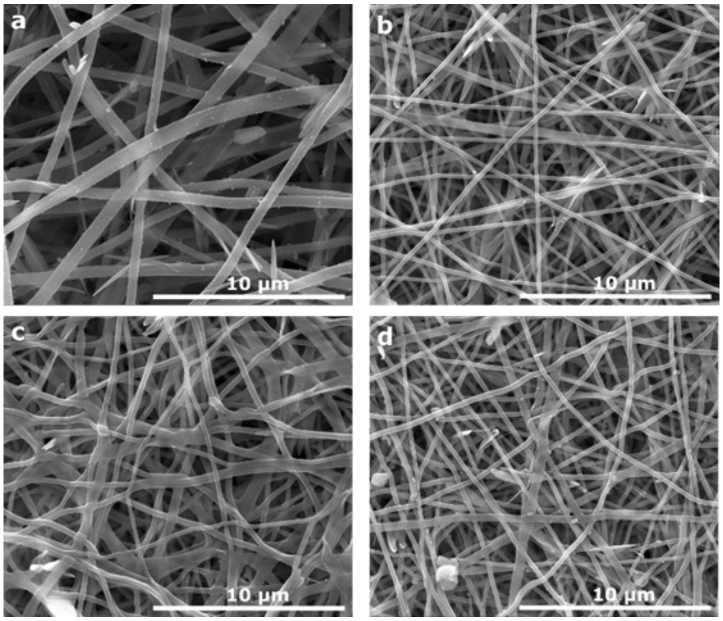

Fig. S1 The as-spun Kraft lignin fiber mats with 0 (a), 10 (b), 30 (c), 50 (d) mol% of $NaNO_3$ in the electrospinning solution.

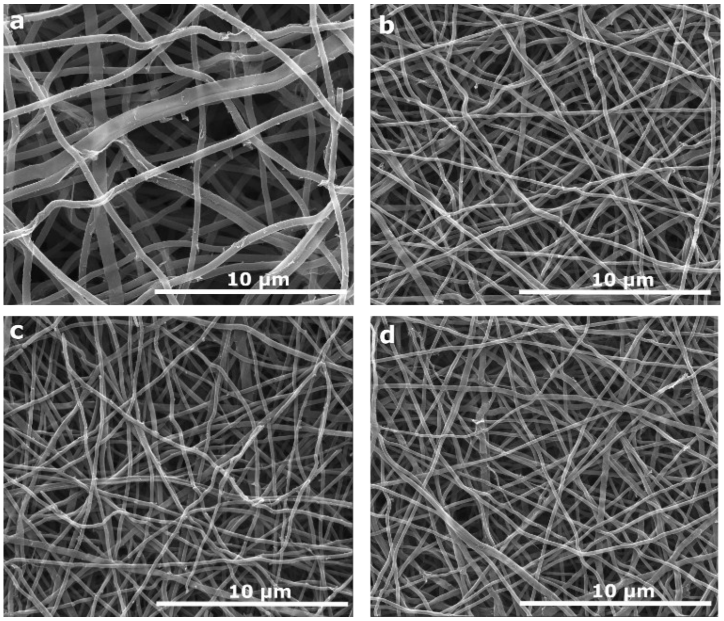

Fig. S2 The CNF mats with 0 (a), 10 (b), 30 (c), 50 (d) mol% of $NaNO_3$ in the electrospinning solution.

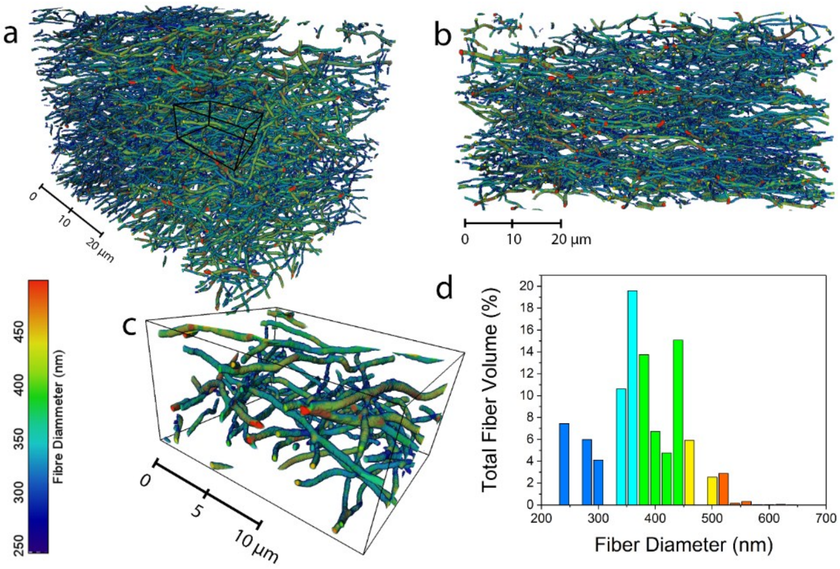

Fig. S3 Fiber diameters of the carbonized N0 fibers, calculated using the local thickness method on the fiber phase shown by applying a colourmap of fiber diameters to the 3D rendering of the fibers (a-c). The yz view in (b) shows no systematic change in the fiber diameter with thickness of the mat (z, time) and a close-up view of the variation of the fiber diameters is shown in (c), taken from a central subvolume of the whole sample (a). The histogram (d) shows the majority of the fibers having a diameter between 300 – 400 nm.

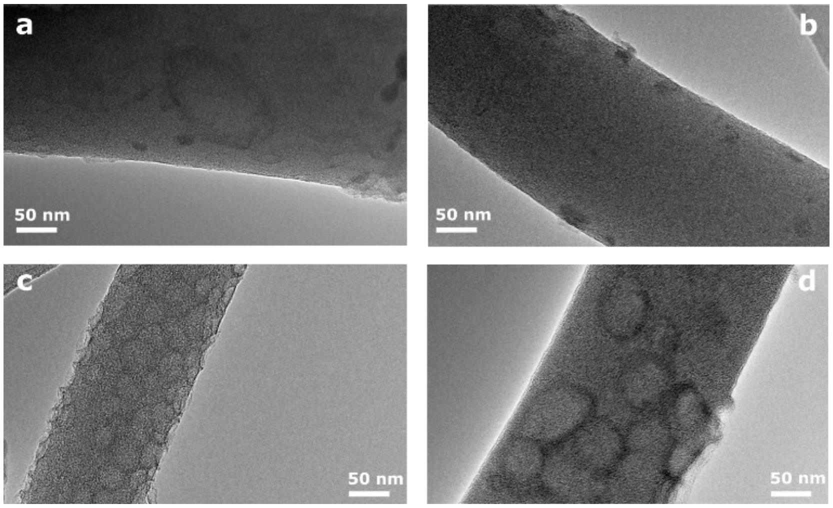

Fig. S4 TEM micrographs of the CNFs with 0 (a), 10 (b), 30 (c), 50 (d) mol% of $NaNO_3$ in the electrospinning solution.

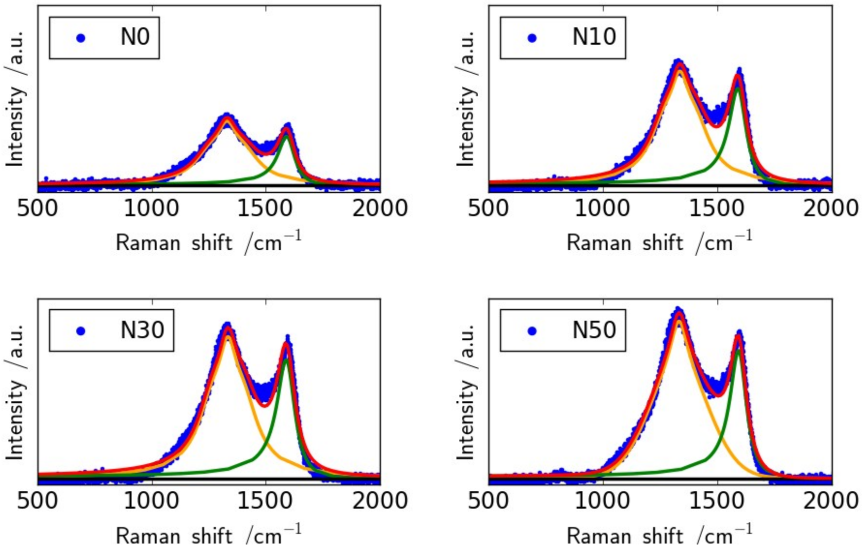

Fig. S5 Normalized Raman spectra of the samples N0, N10, N30, N50 after baseline correction. Each spectrum was fitted using Breit-Wigner-Fano (BWF) and Pseudo-Voigt components.

The peak positions and the intensities (peak heights) were determined by fitting all spectra with a Breit-Wigner-Fano (BWF) and Pseudo-Voigt function using the OriginPro 9.1 suite.

Tab. S4 Fitting parameters of the D- and G-band positions (cm^{-1}) and estimated crystallite size L_a (nm) derived from the Raman spectra.

Sample	D-band	G-band	*I(D)/I(G)*	L_a
N0	1330.8(5)	1595.8(5)	1.313	6.3
N10	1337.2(4)	1595.6(4)	1.179	7.0
N30	1334.9(4)	1594.2(4)	1.195	6.9
N50	1333.4(3)	1597.9(3)	1.229	6.8

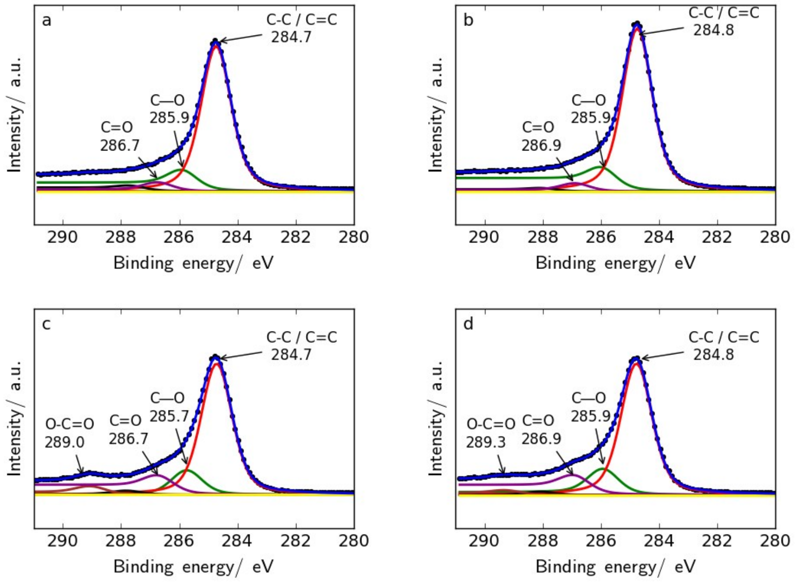

Fig. S6 XPS high-resolution C 1s spectra of (a) N0, (b) N10, (c) N30, (d) N50.

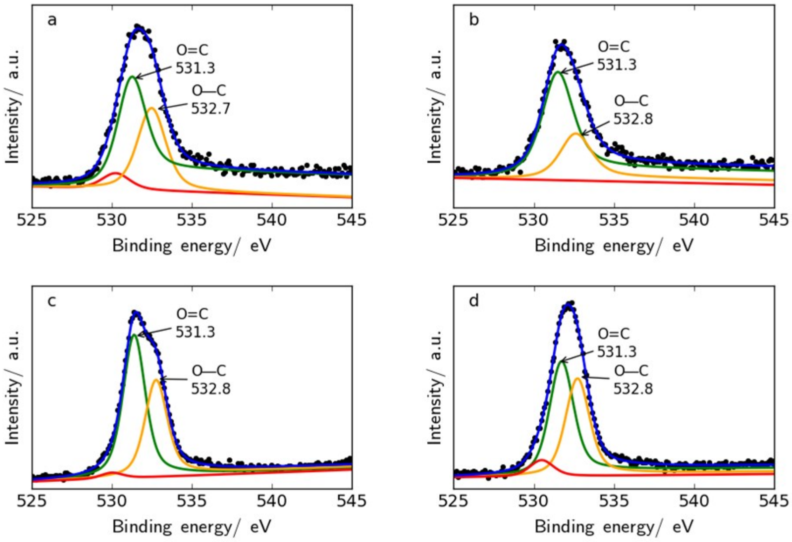

Fig. S7 XPS high-resolution O 1s spectra of (a) N0, (b) N10, (c) N30, (d) N50.

Tab. S5 Amount of CO_2 and CO (μmol g^{-1}) and the resulting oxygen content (wt%) determined by TPD measurements.

Sample	Amount	Amount	Amount	Oxygen

	of CO_2	of CO	of O	content
N0	1095	352	1798	2.9
N10	889	547	1984	3.2
N30	1201	447	2094	3.3
N50	1849	498	2844	4.5

Tab. S6 Overview over data derived from the N_2 and CO_2 gas adsorption experiments.

N_2 adsorption						CO_2 adsorption			
Sample	BET SSA/ $m^2\ g^{-1}$	DFT SSA/ $m^2\ g^{-1}$	Total pore vol./ $cm^3\ g^{-1}$	Pore vol. <2nm/ $cm^3\ g^{-1}$	Pore vol. fraction <2nm/%	DFT SSA/ $m^2\ g^{-1}$	Total pore vol./ $cm^3\ g^{-1}$	Pore vol. <0.8nm/ $cm^3\ g^{-1}$	Pore vol. fraction <0.8nm/%
N0	905	951	0.38	0.32	82.2	1250	0.40	0.24	59.9
N10	972	994	0.46	0.33	72.2	1194	0.39	0.23	60.1
N30	1054	1073	0.48	0.36	76.8	1141	0.34	0.26	77.3
N50	1249	1236	0.52	0.44	84.5	1437	0.48	0.27	55.8

Within the experimental error, a linear correlation with a correlation coefficient of 0.97 between $NaNO_3$ content and DFT surface area derived from the N_2 adsorption could be found. The linear fitting equation is: $s = 5.5695 \cdot c + 938.19$. Where s = DFT surface area [$m^2\ g^{-1}$] an c = concentration of $NaNO_3$ in the electrospinning solution [mol%].

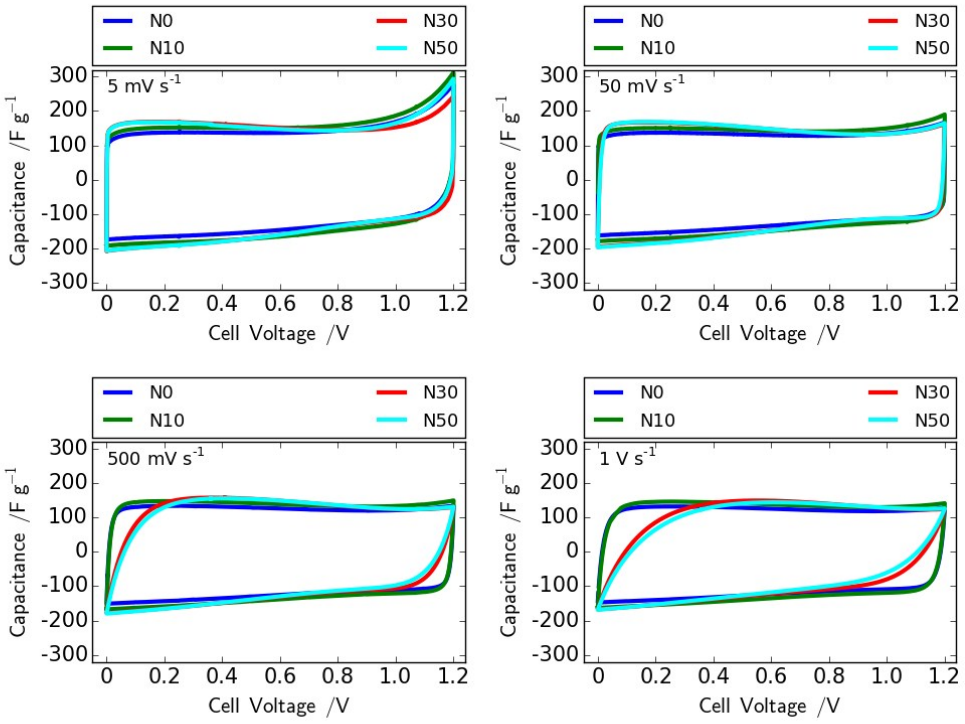

Fig. S8 Voltammograms of N0 (blue), N10 (green), N30 (red), N50 (cyan) measured in 6 M KOH at various scan rates.

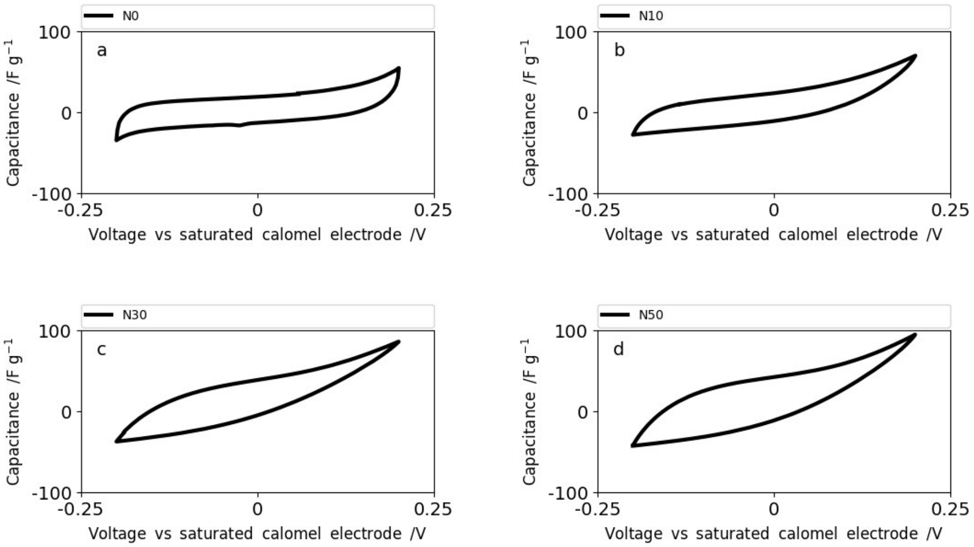

Fig. S9 Voltammograms measured in a three-electrode set-up in 6M KOH.

From the three electrode measurements it is apparent that the capacitive part shifts gradually towards more negative potentials upon addition of $NaNO_3$ compared to N0.

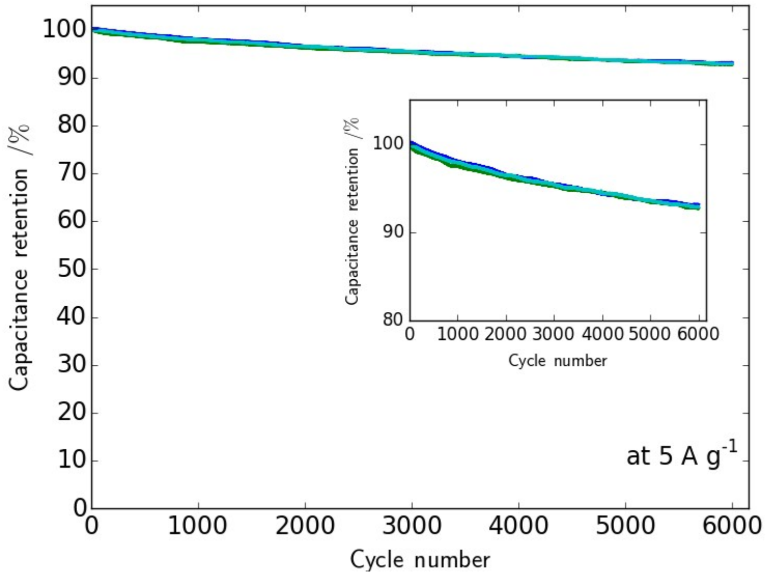

Fig. S10 Capacitance retention for all four samples at 5 A g^{-1} for 6000 cycles.

Capacitance and energy density calculation:

$$C\ [F\ g^{-1}] = \frac{4 * Q * 3.6}{(dV - IR) * m_{cell}}$$

$$C\ [F\ cm^{2}] = \frac{4 * Q * 3.6}{(dV - IR) * A_{WE}}$$

Where:

Q = discharge capacity [mAh] determined by EC Lab software (multiplied by 3.6 for [A*s])

dV= potential difference

IR= IR drop measured from the discharge curves.

m_{cell} = total weight of carbon in the supercapacitor

A_{WE} = are of working electrode (0.785 cm^2)

$$E\ [Wh\ kg^{-1}] = \frac{0.5 * Q * V}{m_{cell}}$$

Where:

Q = discharge capacity

V= potential window

m_{cell} = total weight of carbon in the supercapacitor

Tab. S7 Parameters for the electrospinning process. Some parameters change or are adjusted during the electrospinning process. For this reason, the initial and final value of each parameter is reported here.

	N0		N10, N30, N50		
Parameter	initial	final	initial	final	unit
Humidity	10	10	10	10	%
Temperature	22	22	22	22	°C
Rate	1	1	1.1	1.0	ml h^{-1}
Voltage	19	20	25	24	kV
Distance	20	20	18	18	cm
